# *ABCB6* mRNA and DNA methylation levels serve as useful biomarkers for prediction of early intrahepatic recurrence of hepatitis C virus-related hepatocellular carcinoma

**DOI:** 10.3892/ijo.2013.1854

**Published:** 2013-03-08

**Authors:** RYOUICHI TSUNEDOMI, NORIO IIZUKA, KIYOSHI YOSHIMURA, MICHIHISA IIDA, MASAHITO TSUTSUI, NORIAKI HASHIMOTO, SHINSUKE KANEKIYO, KAZUHIKO SAKAMOTO, TAKAO TAMESA, MASAAKI OKA

**Affiliations:** 1 Departments of Digestive Surgery and Surgical Oncology, Yamaguchi University Graduate School of Medicine, Ube, Yamaguchi 755-8505, Japan; 2 Complementary Medicine, Yamaguchi University Graduate School of Medicine, Ube, Yamaguchi 755-8505, Japan

**Keywords:** ABCB6, hepatocellular carcinoma, prognosis

## Abstract

The poor prognosis of hepatocellular carcinoma (HCC) can be explained largely by the high rate of intrahepatic recurrence (IHR). Identification of genes related to IHR is needed to improve the poor prognosis and important for personalized medicine. Eighty-one HCC specimens were used in this study. We screened for IHR-related genes by DNA microarray analysis. The validation of screening was performed by using real-time PCR. The methylation levels in genomic DNAs were measured by quantitative methylation-specific PCR. Six hepatoma cell lines were used for examination of *ABCB6* expressional regulation. Time-to-event analyses for recurrence after surgery were analyzed by Kaplan-Meier analysis and Cox regression analysis with cutoff values obtained from receiver operating characteristic (ROC) analysis. We confirmed that *ABCB6* mRNA levels were significantly higher in hepatitis C virus (HCV)-related HCCs with early IHR compared to HCV-related HCCs without early IHR (2.5-fold, P=0.01) and the corresponding non-cancerous livers (3.1-fold, P=0.05). Experiments with cell lines showed correlation between DNA methylation and mRNA levels of *ABCB6*. ROC analysis revealed that mRNA levels (0.81 area under the curve, 88% sensitivity and 72% specificity) and DNA methylation levels (0.81 area under the curve, 80% sensitivity and 80% specificity) of *ABCB6* in HCV-related HCCs allowed for the accurate discrimination of the development of early IHR. Cox regression analysis revealed that *ABCB6* mRNA levels was an independent risk factor for IHR of HCV-related HCC. Aberrant mRNA and DNA methylation levels of *ABCB6* may serve as useful predictive biomarkers for early IHR of HCV-related HCC.

## Introduction

Hepatocellular carcinoma (HCC), one of the most lethal malignancies worldwide (1,2), is caused mainly by chronic liver inflammation due to hepatitis C virus (HCV), hepatitis B virus (HBV) and alcohol abuse (1). Despite curative surgical resection and recent advances in treatments, post-surgical recurrence occurs frequently. The poor prognosis of HCC can be explained largely by the high rate of intrahepatic recurrences (IHR) attributable to intrahepatic dissemination of tumor cells (3).

Prediction of IHR could be used to discriminate patients who should receive treatment for the prevention of IHR. Biomarkers for IHR are possible therapeutic targets. Thus, the identification of robust biomarkers that predict IHR of HCC may improve the prognosis of HCC. Many studies have been performed to identify IHR-related biomarkers such as mRNA, microRNA, protein and circulating methylated DNA (4–11). We previously identified *inhibitor of DNA binding 2 (ID2)* as a portal vein invasion-associated gene (12). *ID2* was clearly correlated to disease-free survival time after surgery (13).

Among many factors responsible for IHR, venous invasion, particularly portal vein invasion, is one of the most relevant pathologic factors (14). Recently, cancer stem cells have been considered to largely contribute to carcinogenesis, recurrence and metastasis (15). Cancer stem cells were originally identified in leukemia (16) and then subsequently identified in various solid tumors including HCC (17–22). One cancer stem cell phenotype is chemotherapy resistance largely due to the overexpression of adenosine triphosphate-binding cassette (ABC) transporters (23–25).

In the present study, *adenosine triphosphate-binding cassette, sub-family B (MDR/TAP), member 6* (*ABCB6*) was retrospectively identified as an IHR-related gene by comprehensive analysis. ABCB6 is a mitochondrial ABC transporter involved in iron homeostasis and multi-drug resistance (26–33). We clearly show that the *ABCB6* mRNA and DNA methylation levels were significantly associated with IHR. Epigenetic alterations, including aberrant methylation on CpG islands, affect transcriptional regulation and contribute to carcinogenesis and tumor progression (34–36). In the present study, we found that *ABCB6* expression in hepatoma cell lines was epigenetically regulated by DNA methylation in a CpG island. Our results also indicate that the *ABCB6* mRNA level was an independent risk factor for IHR after curative surgical resection.

## Materials and methods

### Samples

Samples were obtained with informed consent from 81 patients who underwent curative hepatectomy for HCC between May 1997 and July 2006 in the Department of Digestive Surgery and Surgical Oncology, Yamaguchi University Graduate School of Medicine, Japan. The study protocol was approved by the Institutional Review Board for Human Use at Yamaguchi University Graduate School of Medicine. Clinicopathologic features of the 81 HCCs are described in Table I. No patients were undergoing any pre-operative treatment. All patients were followed-up after hepatectomy as reported previously (4). In the present study, we defined IHR up to 2 years after surgery as early IHR, most of which are due to intrahepatic spread of cancer cells (4).

### Hepatoma cell lines

Human hepatoma-derived cell lines Hep 3B, Hep G2, HLE, HuH-6, HuH-7 and SK-HEP-1 were used in this study. These cell lines were purchased from the Health Science Research Resources Bank (Osaka, Japan) and the American Type Culture Collection (Rockville, MD). Cells were cultured in DMEM (Nissui Pharmaceutical, Tokyo, Japan) containing 10% heat-inactivated fetal bovine serum (Life Technologies, Tokyo, Japan) supplemented with penicillin (100 U/ml), streptomycin (100 *μ*g/ml) and sodium bicarbonate (1.5 g/l) at 37°C in 5% CO_2_ in air.

### Semi-quantitative real-time RT-PCR

Semi-quantitative real-time RT-PCR (semi-qRT-PCR) was performed as described previously (13,37) with minor modifications. Real-time PCR amplification was performed by using LightCycler 480 Probe Master (Roche Diagnostics, Tokyo, Japan) and Universal ProbeLibrary Probes (Roche Diagnostics) in a LightCycler System Version 3 (Roche Diagnostics). Primers and probes are listed in Table II. Amplification was performed according to a 2-step cycle procedure consisting of 45 cycles of denaturation at 95°C for 10 sec and annealing/elongation at 60°C for 30 sec. We measured mRNA levels semi-quantitatively by the Δ/Δ threshold cycle (Ct) method. Both the *glyceraldehyde-3-phosphate dehydrogenase (GAPDH)* and *β-actin (ACTB)* genes were used as reference genes. The values are expressed as relative to controls (a mixture of 10 non-tumor liver tissues for clinical samples and HLE cells for cell lines, respectively).

### Quantification of DNA methylation levels at ABCB6 locus

We examined the DNA methylation level by using bisulfite-sequencing and MethyLight (37,38) methods with some minor modifications. Genomic DNA was extracted by using a DNeasy Blood & Tissue kit (Qiagen, Tokyo, Japan) followed by bisulfite treatment with an EZ DNA Methylation-Gold kit (Zymo Research, Orange, CA). Genomic DNAs obtained from cell lines were subjected to bisulfite-sequencing. DNA fragments containing the region from 1.0-kb upstream to 60-bp downstream of the first codon of *ABCB6* were amplified and then sequenced with an ABI 3130XL Genetic Analyzer (Applied Biosystems). Based on the result of bisulfite-sequencing with genomic DNAs of cell lines, we designed primers and probes for MethyLight, a quantitative methylation-specific PCR (qMSP) method (Table II). Real-time PCR amplification was performed as described for semi-qRT-PCR by using a hydrolysis probe and genomic DNA treated with bisulfite. Amplification was performed according to a 2-step cycle procedure consisting of 55 cycles. We measured methylation levels quantitatively with serial dilution of a 100% of the methylated control DNA (EpiTect Control DNA, Qiagen). *Ribonuclease P RNA component H1 (RPPH1)* was used as the internal control. The values are expressed as average of methylation level of *ABCB6* at 2 sites.

### Administration of demethylating agent

The denaturing agent, 5-aza-2′-deoxycitidine (5-aza-dC) (10 *μ*M), was added to the medium. Following a 48-h incubation period, cells were collected and subjected to semi-qRT-PCR analysis.

### Statistical analysis

Data are presented as mean ± standard error in analyses with clinical samples and mean ± standard deviation in analyses with cell lines. Significant differences between two groups were evaluated by the Student’s t-test, Welch’s t-test, or Mann-Whitney U test. Significant differences between three groups were evaluated by analysis of variance (ANOVA) with Kruskal-Wallis test followed by Steel test. Correlation between paired samples was assessed by Spearman rank correlation. Receiver operating characteristic (ROC) analysis was conducted to determine the area under the curve (AUC), sensitivity and specificity. Time-to-event analyses for recurrence after surgery were analyzed by Kaplan-Meier analysis with log-rank test and Breslow test and by using univariable and multivariable (backward stepwise conditional) Cox regression analysis. Calculations were performed by using SPSS Statics 17.0 software (IBM, Tokyo, Japan) and R version 2.13.0 software (the R project website, http://www.r-project.org/). P<0.05 was considered statistically significant.

## Results

### Identification of ABCB6 as an IHR-related gene

**Figure 1:**
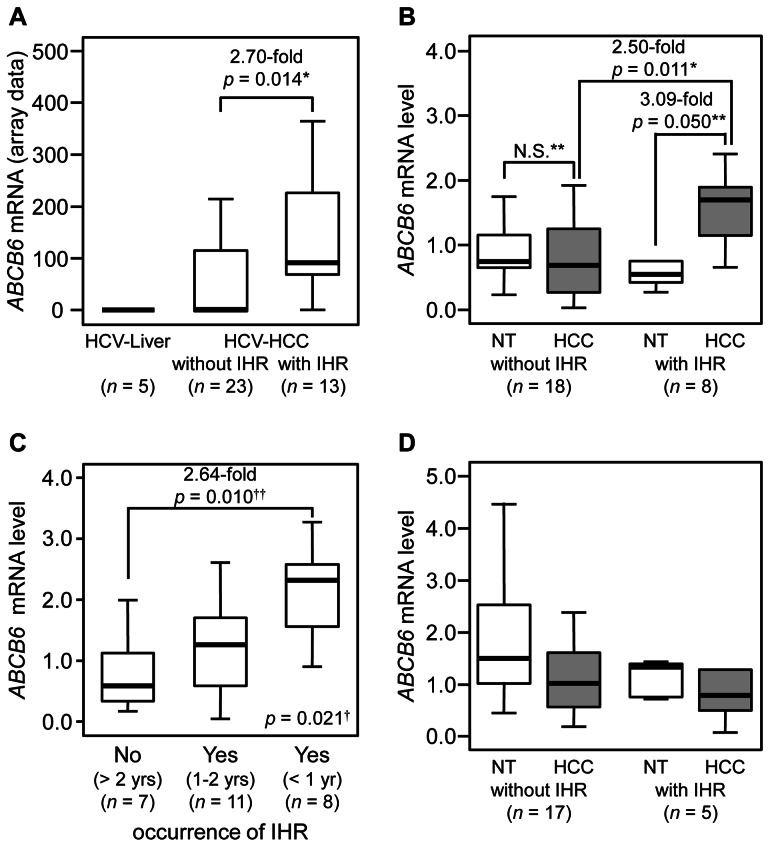
Box and whiskers plots of mRNA levels of *ABCB6* in HCC patients. (A) Data from microarray analysis (comparison in HCV-related HCCs with and without IHR within a year). (B) Validation of the microarray data by semi-quantitative RT-PCR. (C) Patients were divided into 3 groups according to recurrence time after surgery (without recurrence for >2 years, recurrence within 1–2 years and recurrence within a year) and ANOVA was performed. (D) Comparison in non-HCV-related HCC patients. ^*^Mann-Whitney U test; ^**^Wilcoxon signed-rank test for paired samples (non-tumor liver tissues and HCCs in corresponding patients); ^†^ANOVA with Kruskal-Wallis test; ^††^Steel test for multiple comparison.

To identify IHR-related genes, the DNA microarray data obtained previously (12) was examined retrospectively. Nine upregulated genes with both a fold change of ≥2.0 and a Fisher criterion (4,12) of ≥0.6 were found by comparison of HCCs with IHR (n=13) and without IHR (n=23) within a year after surgery (data not shown). Among the upregulated genes, *ABCB6* was identified as an IHR-related gene that had 2.7-fold (P=0.014) higher mRNA levels in HCCs with IHR compared to those in HCCs without IHR (Fig. 1A).

We performed semi-qRT-PCR analysis with newly enrolled samples (n=26) to validate the result from microarray data. The mRNA levels of *ABCB6* in HCCs with IHR within a year after surgery were 2.5-fold (P=0.011) higher than those in HCCs without IHR (Fig. 1B). The remaining 8 upregulated genes identified by microarray were not validated by the semi-qRT-PCR analysis (data not shown). HCCs with IHR within a year showed 3.1-fold (P=0.050) higher *ABCB6* mRNA levels compared to those from corresponding adjacent non-tumor liver tissues (Fig. 1B). We also examined *ABCB6* mRNA levels in HCCs between three groups according to recurrence time (Fig. 1C). These three groups showed a significant difference in the *ABCB6* expression by ANOVA (P=0.021). The *ABCB6* mRNA levels from groups without recurrence (>2 years) and with recurrence (<1 year) represented the lowest and highest values, respectively, and those from the remaining group (with recurrence between 1 and 2 years) were located at the middle. In the cohort of non-HCV-related HCCs, a correlation between the *ABCB6* mRNA level and IHR was not found (Fig. 1D).

### DNA methylation levels of ABCB6 in hepatoma cell lines and clinical samples

We found a CpG island in the 5′-flanking region of *ABCB6* by computational analysis. Genomic DNA from 6 hepatoma-derived cell lines was subjected to bisulfite-sequencing. The CpG island of *ABCB6* was almost fully methylated in HLE and SK-HEP-1 cells (Table III). The remaining 4 cell lines showed low methylation frequencies of CpG sites in the *ABCB6* 5′-flanking region. Highly methylated cells, HLE and SK-HEP-1, had lower mRNA levels of *ABCB6* as compared to the less methylated cell lines (Table III). HLE cells, which harbored the highest DNA methylation level at the *ABCB6* locus, showed the highest *ABCB6* induction level of ∼4.2-fold by the addition of a demethylating agent, 5-aza-dC (Table III). In cell lines, the percentage of DNA methylation of *ABCB6* was inversely correlated with the *ABCB6* mRNA levels (rS = −0.83, P<0.05) and directly correlated with the induction levels (rS = 0.83, P<0.05).

**Figure 2:**
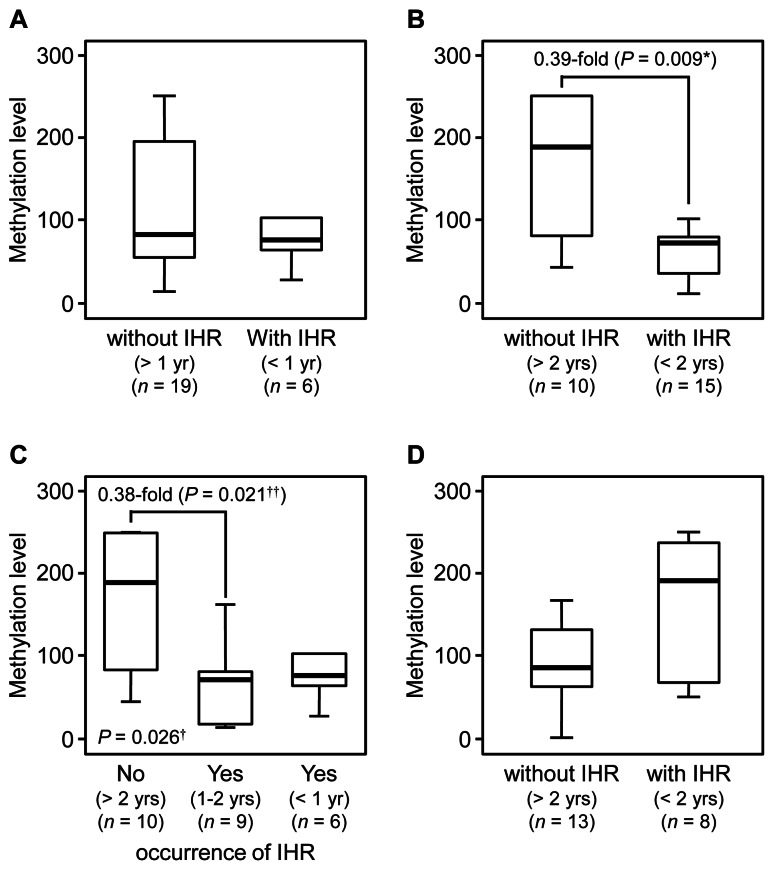
Box and whiskers plots of DNA methylation levels of *ABCB6* in HCC patients. Percentage of methylation level was obtained by quantitative methylation-specific PCR. (A) The *ABCB6* DNA methylation level from HCV-related HCCs with IHR within a year showed no significant difference compared to dose from HCCs without IHR. (B) The *ABCB6* DNA methylation level from HCV-related HCCs with IHR within 2 years was lower than dose from HCCs without IHR. (C) Patients were divided into 3 groups according to recurrence time after surgery and ANOVA was performed. (D) Comparison of non-HCV-related patients (13 patients were without recurrence for >2 years, 2 patients recurred within 1–2 years and 6 patients recurred within a year). ^*^Mann-Whitney U test; ^†^ANOVA with Kruskal-Wallis test; ^††^Steel test for multiple comparison.

In clinical samples, the DNA methylation level of *ABCB6* quantified by qMSP was significantly decreased in HCV-related HCCs with IHR within 2 years after surgery compared to those without IHR (Fig. 2B and C), although the significant difference was not observed with regard to IHR within a year (Fig. 2A). Such difference of the DNA methylation level of *ABCB6* was not seen in non-HCV-related HCCs (Fig. 2D).

### ABCB6 mRNA and DNA methylation levels as indicators of HCC prognosis

**Figure 3:**
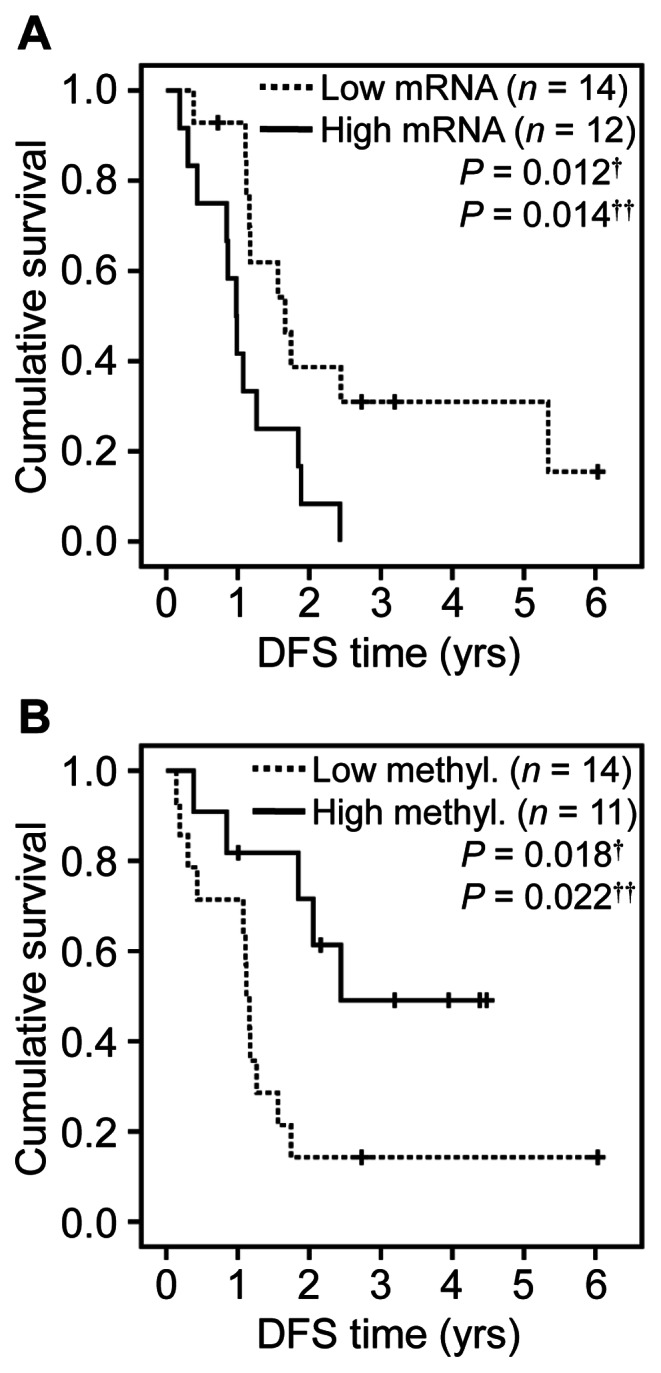
Disease-free survival times of patients with high and low *ABCB6* mRNA and DNA methylation levels. (A and B) Patients with high *ABCB6* mRNA levels and low *ABCB6* methylation levels had a significantly shorter DFS time, respectively. The optimal cutoff values were determined by Youden index that maximized both sensitivity and specificity in receiver operating characteristic curve. ^†^By log-rank test; ^††^by Breslow test.

The cutoff values of *ABCB6* mRNA levels of HCCs were determined by ROC curve for discrimination between HCCs with and without IHR within a year after surgery. The maximum AUC was 0.81 (95% confidence interval, 0.64–0.98) for *ABCB6* mRNA levels, which was followed by 88% sensitivity, 72% specificity and the cutoff value of 1.50. On the basis of the cutoff value, the Kaplan-Meier curve for disease-free survival (DFS) showed that patients with high *ABCB6* mRNA levels had a significantly shorter DFS time than those with low *ABCB6* mRNA levels (P= 0.012, Fig. 3A). In the cohort of HBV-related HCCs, a correlation between the *ABCB6* mRNA level and DFS time was not found (data not shown).

In the ROC curve for discrimination between HCCs with and without IHR within 2 years after surgery, the maximum AUC was 0.81 (95% confidence interval, 0.63–1.00) for DNA methylation levels of *ABCB6*. On the basis of the ROC curve, the optimal cutoff value of DNA methylation level of *ABCB6* was determined to be 82.2%, which corresponded to a performance with 80% sensitivity and 80% specificity. Patients with low DNA methylation levels at the *ABCB6* locus had a significantly shorter DFS time than those with high DNA methylation levels at the *ABCB6* locus (P=0.018, Fig. 3B).

**Figure 4:**
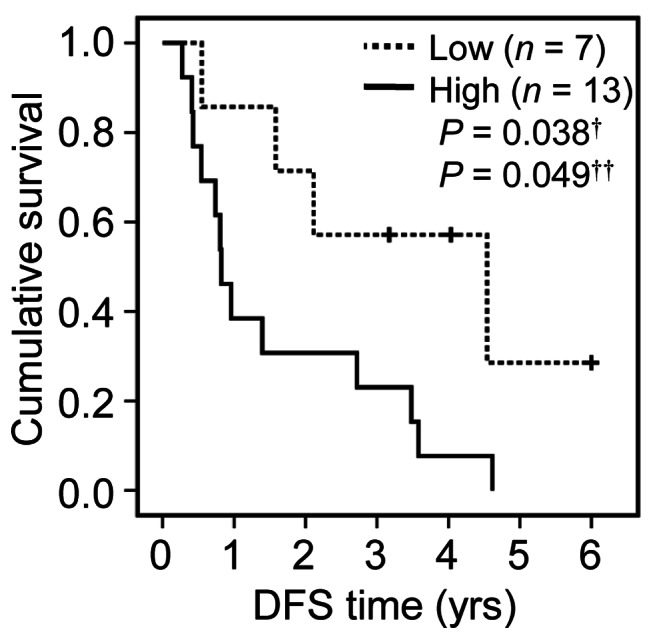
Disease-free survival times of newly enrolled patients with high and low *ABCB6* mRNA levels. Patients with high *ABCB6* mRNA levels had a significantly shorter DFS time in newly enrolled HCV-related HCC patients again. Used cutoff value is the same as the value used in Fig. 3A. ^†^By log-rank test; ^††^by Breslow test.

Finally, we performed Cox regression analysis of recurrence after surgery in HCV-related HCC patients to determine the independent hazard risk factor (Table IV). In addition to mRNA and DNA methylation levels of *ABCB6*, tumor histological differentiation grade, UICC tumor stage and number of primary tumors were examined as variables, because Kaplan-Meier curves for DFS showed significant differences (data not shown). The univariate analysis showed that *ABCB6* mRNA level, *ABCB6* DNA methylation level and tumor histological grade were significant risk factors for IHR after surgery. The multivariate analysis using backward stepwise likelihood ratio revealed that the *ABCB6* mRNA level was an independent risk factor for IHR after surgery. Furthermore, using different HCC patients (n=20) from the patients enrolled into the identification of the IHR-related gene, Kaplan-Meier curves for DFS showed significant difference again (P=0.04, Fig. 4). In this validation cohort, the performance for discrimination between HCC patients with and without IHR within a year after surgery was 89% sensitivity, 55% specificity, 86% positive predictive value and 62% negative predictive value.

## Discussion

In this study, we identified the *ABCB6* gene as an IHR-related gene by comprehensive analysis. Because virus type and differentiation grade largely influence the expression profile in HCCs (39,40), we restricted the samples for DNA microarray analysis data to patients with HCV and moderately or poorly differentiated HCCs. Indeed, the correlation of IHR and *ABCB6* was seen in HCV-related HCCs, but not seen in non-HCV-related HCCs. Unfortunately, the *ABCB6* gene was the only gene validated by semi-qRT-PCR analysis. Perhaps most genes which passed the microarray analysis were not validated by semi-qRT-PCR analysis due to not only the difference in patient population but also the difference in the normalization in each analysis. While *GAPDH* and *ACTB* were used as reference genes in the PCR analysis, normalization from all gene expressions was used instead of the specific reference gene in the microarray analysis. Indeed, the *GAPDH* mRNA levels in clinical samples represented by the normalized microarray data varied (data not shown).

We found that the *ABCB6* mRNA levels in primary HCV-related HCCs that recurred within a year were significantly higher than those in HCV-related HCCs that did not recur within a year (Fig. 1A–C). Such a finding is HCV-specific, it was not observed in non-HCV-related HCCs (Fig. 1D). Furthermore, consistent with previous reports (41,42), the *ABCB6* mRNA levels were increased in HCCs compared to those in adjacent non-tumor liver tissues. The *ABCB6* gene encodes a mitochondrial ABC transporter involved in iron homeostasis, mitochondrial respiratory function, stability of mitochondrial DNA and multidrug resistance (26,27). Multi-drug resistance caused by overexpression of ABC transporters is a phenotype of cancer stem cells, which are thought to be important in metastasis and recurrence (15). In this study, HuH-7 cells showed high mRNA and low DNA methylation levels of *ABCB6* and oppositely, HLE and SK-HEP-1 cells showed low mRNA and high DNA methylation levels of *ABCB6* (Table III). It was reported that HuH-7 cells contained cancer stem cells, but HLE and SK-HEP-1 cells contained no cancer stem cells (22,43,44). We have preliminary data that *ABCB6* expression is more significantly increased in cancer stem cell-like sphere cells as compared to other ABC transporters (unpublished data). There is a possibility that the HCCs overexpressing *ABCB6* harbor cancer stem cells responsible for intrahepatic metastasis.

Iron, which is one of the substrates of ABCB6, plays a crucial role in proliferation and DNA synthesis and neoplastic cells have an increased requirement for iron (45). It has been suggested that changes in iron regulation characterize the malignant state and the iron regulatory gene signature was associated with breast cancer prognosis (46). Recent loss of function and gain of function analysis with hepatoma cell line HuH-7 revealed that *ABCB6* plays a role in cell growth and proliferation by targeting the cell cycle (41). An iron chelator, deferoxamine, exerts its antiproliferative effect by cell cycle arrest and induction of apoptosis (47). Recently, it was reported that deferoxamine was an effective treatment for advanced HCCs that did not respond to anti-tumor drugs including sorafenib (48).

Our results suggest that ABCB6 expression is regulated epigenetically at least partially, as *ABCB6* mRNA and DNA methylation levels are inversely correlated in hepatoma cell lines (Table III). Genome-wide hypomethylation and gene-specific hypermethylation of promoter regions are well-known abnormalities in cancer (49). Genome-wide alterations of histone H3 lysine 9 dimethylation also affect the *ABCB6* mRNA level (50). As another possible form of *ABCB6* regulation, some genes encoding ABC transporters were upregulated by downregulation of microRNAs in HCC (42). In this study, although significant *ABCB6* mRNA overexpression was observed in HCCs from patients with IHR within a year but not within 1 to 2 years, significant decreased DNA methylation levels of *ABCB6* was already observed in HCCs from patinets with IHR within 1 to 2 years (Figs. 1C and 2C). Furthermore, the inverse correlation between the mRNA and DNA methylation levels of *ABCB6* was only seen in a subset population which consists of patients with IHR within a year after surgery (data not shown). In addition to DNA demethylation, abberations of other factors such as microRNAs would also be needed for *ABCB6* overexpression.

Time-to-event analysis revealed that the *ABCB6* mRNA and DNA methylation levels were significantly correlated to DFS time (Figs. 3 and 4). The relationship between tumor *ABCB6* mRNA level and shorter DFS time was specific for HCV-related HCC but not HCV-unrelated HCC (data not shown). Cox regression analysis provided a novel finding that tumor *ABCB6* mRNA levels can be an independent risk factor for IHR of HCC after surgery (Table IV). The DNA methylation levels at the *ABCB6* locus were related to IHR within 2 years but not 1 year after surgery, the DNA methylation level of *ABCB6* may be insufficient as an early predictor for intrahepatic recurrence.

In conclusion, the *ABCB6* status, which consists of levels of mRNA and DNA methylation, is a useful biomarker for the prediction of recurrence of HCV-related HCCs due to intrahepatic metastasis and *ABCB6* is also a possible target gene for therapy. Further gene-targeting experiments are needed to disclose the role of *ABCB6* in IHR of HCC, especially with regard to the role of cancer stem cells in metastasis.

## Figures and Tables

**Table I:** Clinicopathological features of 81 patients used in this study.

	HCC patients		
Clinicopathological feature	Total (N=81)	HCV-related (n=53)^b^	Non-HCV-related^a^ (n=28)^c^
Gender			
Male	62	40	22
Female	19	13	6
Age (years)^d^	66.0±1.3	68.2±0.8	61.8±2.9
Tissues surrounding HCC			
Normal liver	5	2	3
Chronic hepatitis	36	25	11
Liver cirrhosis	40	26	14
AFP (ng/ml)^d^	1,014.4±339.7	1,172.3±394.1	721.2±350.9
Tumor size (cm)^d^	3.9±0.3	3.8±0.2	4.2±0.6
Primary tumor			
Single tumor	49	30	19
Multiple tumors	32	23	9
Tumor histological grade			
Well-differentiated	20	12	8
Moderate-differentiated	51	34	17
Poorly-differentiated	10	7	3
UICC stage			
I	29	22	7
II	47	29	18
IIIA–IV	5	2	3

a HBV-related HCC patients (n=18) and both HBV and HCV-unrelated patients (n=10).

b Among 53 HCV-related patients, 18 patients were overlapped with mRNA and DNA analysis (mRNA analysis, n=26; DNA analysis, n=25; mRNA analysis for validation of time-to-event analysis, n=20).

c Among 28 non-HCV-related patients, 15 patients were overlapped with mRNA and DNA analysis (mRNA analysis, n=22; DNA analysis, n=21).

d Represented values were mean ± standard error.

**Table II:** Used primers and hydrolysis probes in this study.

	Sequence (5′→3′)
For PCR analysis	
ABCB6	
5′-primer	GGACCAAGATGTGGAAAGGA
3′-primer	CCAAAATCTCGCCAGGTAGA
Hydrolysis probe	UPL probe no. 66^a^
GAPDH	
5′-primer	AGCCACATCGCTCAGACAC
3′-primer	GCCCAATACGACCAAATCC
Hydrolysis probe	UPL probe no. 60^a^
ACTB	
5′-primer	CCAACCGCGAGAAGATGA
3′-primer	CCAGAGGCGTACAGGGATAG
Hydrolysis probe	UPL probe no. 64^a^
For qMSP analysis^b^	
ABCB6-MSP1 (position: −327 to −207)	
5′-primer	GGGGTTATAGTcgTGGAGc
3′-primer	AAAACAcgTAcgCcgTCT
Hydrolysis probe	FAM-GTGGGTTTGTAGTTGGTAGGAGGGTT-BHQ
ABCB6-MSP2 (position: −710 to −607)	
5′-primer	TAGATTTTTTGTTGTTTcgc
3′-primer	TCTAAAcgAcgACCTAAACA
Hydrolysis probe	FAM-AAGAGAAATGGGATGGGGATTTTG-BHQ
RPPH1	
5′-primer	AATGAGGTGTAGAAGGTTGATGGT
3′-primer	CATAATTAAATCACTTCCCACCAAA
Hydrolysis probe	UPL Probe no. 10^a^

a Universal ProbeLibrary (Roche Applied Bioscience).

b CpG sites are presented as lower case letters. Position was given relative to the first codon of *ABCB6*. Start site of the first exon of *ABCB6* is positioned at −317.

**Table III:** *ABCB6* DNA methylation and mRNA levels in hepatoma cell lines.

		mRNA level^a^			
Cell lines	% DNA methylation^b^	5-aza-dC		Ratio (with/without)	P-value
		Without	With		
HLE	98.2	1.0±0.1	4.2±0.5	4.2-fold	<0.05
SK-HEP-1	86.6	25.0±1.1	47.0±4.3	1.9-fold	<0.05
Hep 3B	37.2	161.0±7.5	265.8±15.0	1.7-fold	<0.05
HuH-6	34.1	179.2±13.7	252.9±29.9	1.4-fold	<0.05
HuH-7	30.5	150.8±12.5	228.5±17.7	1.5-fold	<0.05
Hep G2	28.7	720.3±38.1	1,031.1±101.1	1.4-fold	<0.05

a mRNA levels were measured by semi-qRT-PCR as described in Materials and methods. *ABCB6* mRNA levels in HLE cells without 5-aza-dC was set to 1.0. Represented values were mean ± standard deviation. P-values were obtained by Student’s t-test or Welch’s t-test.

b DNA methylation levels at 5′-flanking region of *ABCB6* (∼1.0-kb length containing 82 CpG sites) were examined by bisulfite-sequencing.

**Table IV:** Cox regression analysis of IHR after surgery in HCC patients.

	Univariate analysis		Multivariate analysis	
	
Risk factor	HR (95% CI)	P-value	HR (95% CI)	P-value
*ABCB6* mRNA (low vs high)	3.03 (1.22–7.52)	0.017	3.21 (1.10–9.34)	0.033
*ABCB6* methylation (low vs high)	0.29 (0.10–0.86)	0.025		
Tumor diff. grade (well vs moderate-poor)	3.86 (1.31–11.45)	0.015		
Stage of UICC (I vs II–IIIA)	1.81 (0.78–4.19)	0.168		
Primary tumor (single vs multiple)	1.43 (0.60–3.39)	0.422		

HR, hazard risk. CI, confidential interval. Diff., histological differentiation.
